# Realizing UWB Antenna Array with Dual and Wide Rejection Bands Using Metamaterial and Electromagnetic Bandgaps Techniques

**DOI:** 10.3390/mi12030269

**Published:** 2021-03-06

**Authors:** Ayman A. Althuwayb, Mohammad Alibakhshikenari, Bal S. Virdee, Pancham Shukla, Ernesto Limiti

**Affiliations:** 1 Department of Electrical Engineering, College of Engineering, Jouf University, Sakaka, Aljouf 72388, Saudi Arabia; 2 Electronic Engineering Department, University of Rome “Tor Vergata”, Via del Politecnico 1, 00133 Rome, Italy; limiti@ing.uniroma2.it; 3 Center for Communications Technology, London Metropolitan University, London N7 8DB, UK; b.virdee@londonmet.ac.uk (B.S.V.); p.shukla@londonmet.ac.uk (P.S.)

**Keywords:** bandgap rejection, electromagnetic bandgap (EBG), metamaterials (MTM), composite right/left-handed structures (CRLH), ultra-wide band (UWB), antennas

## Abstract

This research article describes a technique for realizing wideband dual notched functionality in an ultra-wideband (UWB) antenna array based on metamaterial and electromagnetic bandgap (EBG) techniques. For comparison purposes, a reference antenna array was initially designed comprising hexagonal patches that are interconnected to each other. The array was fabricated on standard FR-4 substrate with thickness of 0.8 mm. The reference antenna exhibited an average gain of 1.5 dBi across 5.25–10.1 GHz. To improve the array’s impedance bandwidth for application in UWB systems metamaterial (MTM) characteristics were applied it. This involved embedding hexagonal slots in patch and shorting the patch to the ground-plane with metallic via. This essentially transformed the antenna to a composite right/left-handed structure that behaved like series left-handed capacitance and shunt left-handed inductance. The proposed MTM antenna array now operated over a much wider frequency range (2–12 GHz) with average gain of 5 dBi. Notched band functionality was incorporated in the proposed array to eliminate unwanted interference signals from other wireless communications systems that coexist inside the UWB spectrum. This was achieved by introducing electromagnetic bandgap in the array by etching circular slots on the ground-plane that are aligned underneath each patch and interconnecting microstrip-line in the array. The proposed techniques had no effect on the dimensions of the antenna array (20 mm × 20 mm × 0.87 mm). The results presented confirm dual-band rejection at the wireless local area network (WLAN) band (5.15–5.825 GHz) and X-band satellite downlink communication band (7.10–7.76 GHz). Compared to other dual notched band designs previously published the footprint of the proposed technique is smaller and its rejection notches completely cover the bandwidth of interfering signals.

## 1. Introduction

Ultra-wideband (UWB) systems enable high data rate wireless transmission and therefore are not just restricted to wireless communications systems but have application in radar and imaging systems because they enable acquisition of high-resolution images. UWB antennas are an essential component of UWB systems providing low power dissipation and large impedance bandwidth. Characteristics sought after in UWB antennas include small form factor, desired radiation characteristics, and cost effectiveness [1,2]. 

The UWB spectrum is shared with other narrowband services, for example, wireless local area network (WLAN) for IEEE 802.11a in the USA (5.15–5.35 and 5.725–5.825 GHz), which can interfere with UWB systems [3]. Therefore, it is important to eliminate such signals from affecting the performance of UWB systems. This is normally achieved by using filters in the RF front-end. Although filters are effective devices, they can introduce extra loss and cost as well as affect the overall size of the systems [4,5]. To overcome interference between UWB systems and narrowband systems that operate within the UWB spectrum extensive research has been conducted over the past several years on developing UWB antennas that possess band-reject characteristics. Various band-notched UWB antennas have been reported using different design techniques [6,7,8,9,10,11,12,13,14,15,16,17,18]. 

Previous examples of band-notched UWB antennas include reference [19], where two closely spaced but extremely narrow band notches are achieved by adding two stubs on each side of the patch antenna, whose lengths are quarter-guide wavelength at the desired notching frequency. Electromagnetic (EM) analysis reveals that strong rejection is realized when adjacent currents flow in opposite directions and cancel the radiated fields. In fact, at the notch frequencies, the current flowing on the stubs is in opposite direction (out-of-phase) from the current on the antenna edges. In [20], a notched band is realized by placing electromagnetic bandgap (EBG) structures on the coplanar waveguide (CPW) feedline. The EBG structures are constructed on the opposite face of the substrate and aligned with the CPW feedline. Shorting pins are used to connect the EBG structures with the feedline. Essentially, the CPW feedline uses the EBG structures as a ground-plane. The resonant frequencies of the EBG structures are tuned so that they merged to create a rectangular shaped notched band. In [21], a dual-band notch is realized by loading a pair of split-ring resonators (SRR) on the opposite side of the CPW-fed monopole. The SRRs are placed under the feedline. Dimensions of the SRR determine the notching frequency. The pair of SRRs are separated by quarter-guided wavelength to avoid mutual coupling effects. In [22], strategically placing a stepped slot, the antenna can be excited under differential-mode of operation. Dual notched bands are created by introducing two pairs of quarter–wavelength slits, a pair of half-wavelength stubs, and a half-wavelength stub. The bandwidths of the dual notched bands can be controlled by adjusting the lengths of the stubs and slits. In [23], band notch functionality is realized by using two pairs of horizontally placed folded-strip resonators with an inductive coupling scheme. Finally, in [24], a stepped slot antenna is used to realize UWB impedance matching characteristics. By slitting an open-ended quarter wavelength slot and a short-ended half-wavelength ring slot on the ground near the stepped slot, a notched band of 5.15–5.85 GHz is created. The single and dual notched band UWB antennas described above have a relatively narrow bandwidth. Antennas are needed to suppress interference signals from not just narrowband signals but also wideband signals such as the X-band downlink satellite communication band (660 MHz). Therefore, it is necessary to investigate techniques to realize wide rejection bandwidth UWB antennas. 

This article describes a technique to realize an UWB antenna array that has two wide rejection bands to eliminate interference signals from unwanted wireless systems that operate in the ultra-wideband frequency domain. This is accomplished by applying metamaterial (MTM) and EBG techniques. Application of MTM extends the operationally bandwidth of the array, and EBG is used to create notched bands at the interfering frequencies. The simulation results of the design were verified using two different 3-D full-wave electromagnetic tools.

## 2. Antenna Array Structure

### 2.1. Reference Antenna Array

**Figure 1:**
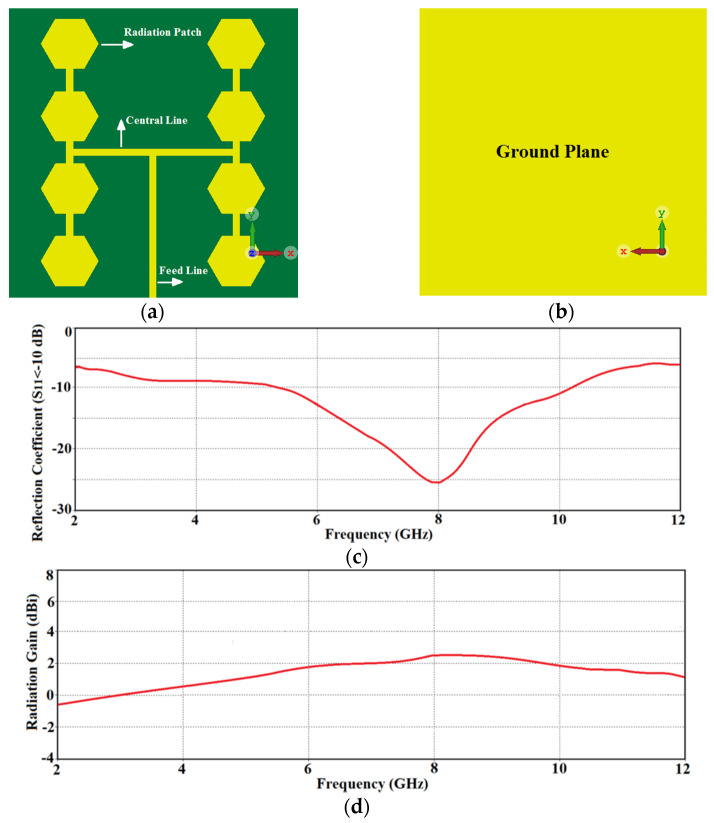
Geometry of the reference antenna: (**a**) top-side of substrate, (**b**) back-side of substrate (ground-lane), (**c**) simulated reflection-coefficient response, and (**d**) simulated radiation gain response.

The configuration of the reference antenna array shown in Figure 1a,b consists of a 2 × 4 matrix of hexagonal patches that are interconnected to each other with a high impedance microstrip-line. The bottom side of the substrate is a ground-plane as shown in Figure 1b. The array is excited through a common central feedline. The patches in the array are based on a standard design and its performance was simulated and optimized using CST Microwave Studio. The array was fabricated on the FR-4 substrate with dielectric constant of 4.3, tanδ of 0.025, and thickness of 0.8 mm. The thickness of the conductive layer made of copper is 0.035 mm. The total dimensions of the antenna are 20 mm × 20 mm × 0.87 mm. Geometrical parameters of the optimized array are listed in Table 1. The reflection-coefficient response of the reference antenna array in Figure 1c shows that it operates over 5.25 GHz to 10.1 GHz corresponded to 63.19% practical bandwidth, which falls short for UWB systems defined between 3.1 GHz to 10.6 GHz. Figure 1d show the average radiation gain of the array across its operating band is 1.5 dBi. 

### 2.2. Fabrication Process 

**Figure 2:**
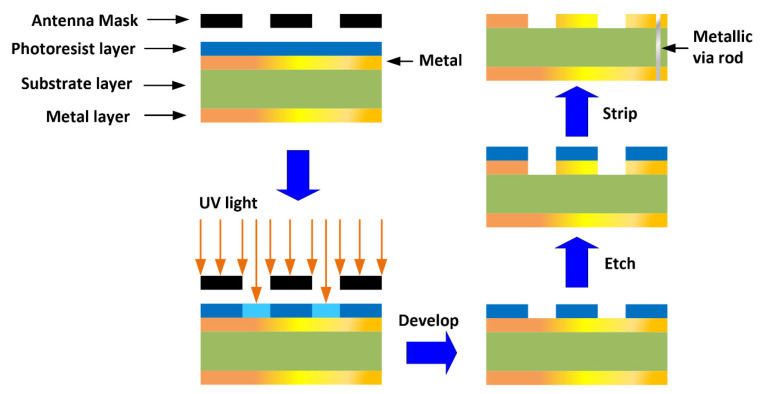
Photo-lithographic fabrication process to implement the antenna.

The fabrication of the antenna is made by standard photo-lithographic method based on a chemical etching process as illustrated in Figure 2. This involves the removal of the unwanted conductive regions of the metallic layer. The photo-lithographic process uses light to transfer the antenna pattern from a photomask to a photosensitive chemical photoresist on the substrate. The thickness of the photoresist layer is typically 1 micron. A series of chemical treatments is performed to etch the exposure pattern on the metal. This procedure is comparable to a high precision version of the method used to make printed circuit boards. Precision of this technique is typically down to less than 2 microns and the repeatable accuracy is ±10 microns, which means that the fabrication method will have negligible effect on the performance of the antenna whose dimensions are specified in Table 1. The vias are created using the standard through-hole technology. This involves drilling a hole through the dielectric substrate which then needs to be cleaned of debris from the drilling operation before a conductive wire is inserted through it. The wires are cut to size and both ends of the wire are soldered to the microstrip surface to create an electrical short circuit. Alternately, through-hole plating process can be used to metallize the via-holes. Through-hole plating involves chemically coating the holes with a thin layer of copper through a process called electroless copper deposition. This gives the copper plating a base to build up from. The full amount of copper is then electroplated in the via holes by connecting the board to an electrical charge so that the boards act as cathodes for the electroplating process. 

In the next section, the composite right/left-handed (CRLH) structure is applied in the antenna structure to incorporate metamaterial characteristics to extend the arrays impedance bandwidth. 

### 2.3. Transforming the Antenna Array for Ultra-Wideband (UWB) Operation 

**Figure 3:**
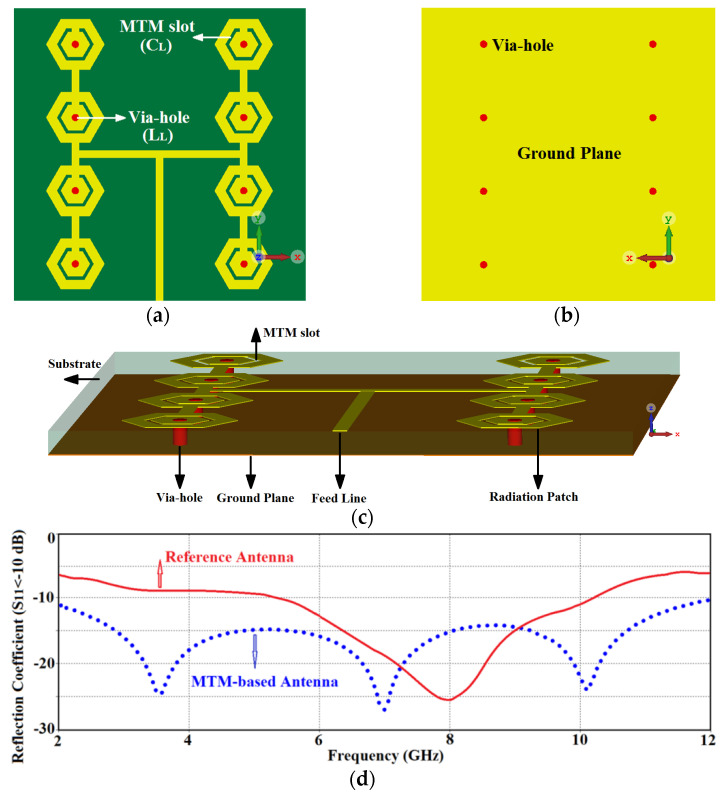

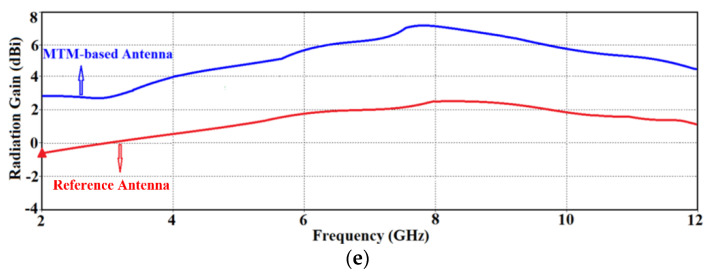
Configuration of the proposed metamaterial (MTM) based antenna array: (**a**) top-side view of substrate, (**b**) back-side view of substrate (ground-plane), (**c**) isometric view showing all the structural components and their locations, (**d**) reflection-coefficient response of the reference and MTM antenna array, and (**e**) radiation gain of the reference and MTM antenna array.

The bandwidth of the reference antenna array defined for S11≤−10 dB in Figure 1 is between 5.25 GHz to 10.1 GHz. This partially covers the required UWB spectrum defined by the American Federal Communications Commission (FCC) to be between 3.1 GHz to 10.6 GHz. Therefore, it was necessary to extend the array’s bandwidth to fulfill the UWB bandwidth criteria. This was achieved by incorporating CRLH structure in the hexagonal patches of the reference antenna array. This involved etching a hexagonal slot in the radiating patch and inserting a metallic via at the center of the patch to ground it, as shown in Figure 3a–c. Geometrical parameters of the optimized array are listed in Table 2. The hexagonal slot in the radiating patch essentially acts like a series left-handed capacitance, and the metallic via acts like a shunt left-handed inductance. Moreover, surface currents that flow over the antenna establish parasitic right-handed capacitance between the patch and the ground-plane, and series right-handed inductance on its surface. This modification essentially introduces metamaterial characteristics in the reference antenna. The consequence of this is significant improvement of the impedance bandwidth as well as the radiation gain over the UWB spectrum as is confirmed by the simulation results in Figure 3d,e. The reference antenna’s impedance bandwidth for S11≤−10 dB is extended from between 5.25 GHz and 10.1 GHz to between 2 GHz and 12 GHz, which corresponds to a fractional bandwidth of 142.85%. Compared to the reference array this constitutes a bandwidth improvement of ~80%. The corresponding average gain improved from 1.5 dB to 5 dBi. 

### 2.4. MTM Based UWB Antenna Array with Wide Rejection Bands

**Figure 4:**
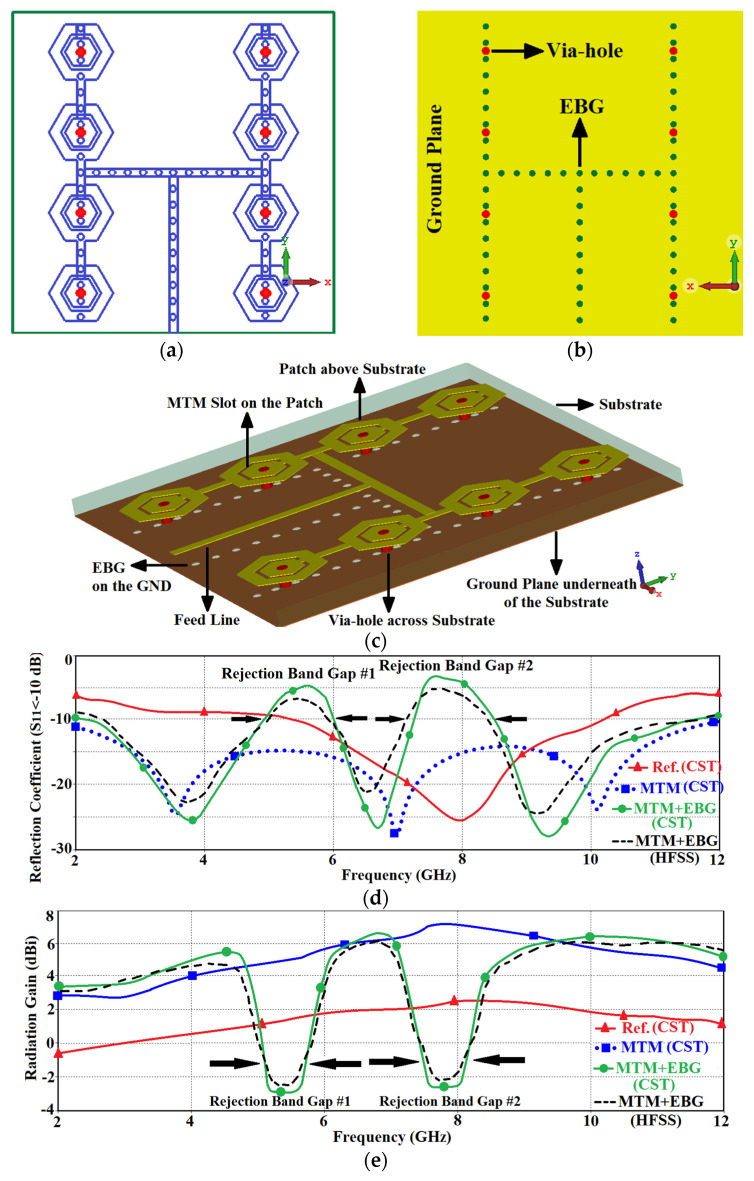
Layout of the proposed MTM based antenna array loaded with electromagnetic bandgap (EBG) slots: (**a**) top-side of substrate, (**b**) back-side of substrate (ground-plane), (**c**) isometric view showing the structural components and their locations, (**d**) reflection coefficient response comparison of the reference, MTM, and MTM with EBG loading, and (**e**) radiation gain of the reference, MTM, and MTM with EBG loading.

As the UWB frequency band is shared with other wireless systems such as WLAN operating across 5.150–5.825 GHz it can experience interference. To prevent this the RF front-end of UWB systems require a filter which can be costly and affect the size of the system. To overcome the effects of interfering signals various techniques have been previously proposed [3,19,20,21,22,23,24] that introduce notching function on the UWB antenna; however, their rejection band is narrow to completely reject wideband interfering signals. Dual wideband rejection band is introduced here by applying electromagnetic bandgaps on the back side of the MTM antenna array. This technique does not affect the array’s physical dimensions. The proposed EBG is realized by implementing arrangement of circular slots on the antenna’s ground-plane under the patches and interconnecting microstrip-lines, as shown in Figure 4a–c. The geometrical parameters of the proposed antenna array are given in details in Table 3. The exact positions of the EBG slots were optimized using CST Microwave Studio. The results in Figure 4d show the two notched bands are highly selective and realized between 4.90–6.04 GHz and 7.09–8.50 GHz. Therefore, the proposed antenna array is capable of suppressing interference affects from WLAN (5.15–5.825 GHz) and X-band satellite downlink communication band (7.10–7.76 GHz). Two different 3-D full-wave electromagnetic simulation tools were used to validate the design, that is CST Microwave Studio and HFSS. There is excellent agreement between both tools which confirms the feasibility of the proposed technique. 

## 3. State-of-the-Art Comparison

The proposed MTM-EBG technique to realize UWB antenna array with dual notched band characteristics is compared with other single and dual notched band UWB antennas in Table 4. It is evident that the proposed antenna has a smaller footprint compared to single and dual notched band antennas in references [19,20,21,22,23]. The notched band of the proposed antenna is significantly larger than any of the prior designs. Moreover, unlike other dual notched band antennas its EBG frequency can be controlled, and its design and implementation are relatively easy. Also, the proposed antenna is excited with a single feed port unlike other cited works that need two-port excitations except for reference [3]. As a result, the characteristics of the proposed MTM-EBG UWB antenna array and its simplicity make it a viable candidate for various UWB applications. 

## 4. Conclusions

Feasibility of a low-profile UWB antenna array design with two highly selective wide rejection bands has been investigated. This was achieved by introducing metamaterial characteristics in the hexagonal patch antenna to extend its operational bandwidth. Dual notched bands were incorporated in the design to prevent the UWB system being affected by interference from WLAN (5.15–5.825 GHz) and X-band satellite downlink communication band (7.10–7.76 GHz). This was accomplished by loading the ground-plane of the antenna with electromagnetic bandgap slots. The proposed techniques had no effect on the dimensions of the antenna array. Unlike previous dual-notched band designs the proposed antenna is smaller and has a wider notched band characteristic to eliminate interfering signals. 

## Figures and Tables

**Table 1:** Geometrical parameters of the reference antenna array.

Parameters	Dimensions (mm)
Radius of hexagonal patches	2
Width of connecting lines	0.5
Horizontal gap between patches	7.5
Vertical gap between patches	1.55
Length of the central feedline	10
Width of the feedline	0.5

**Table 2:** Geometrical parameters of the MTM inspired ultra-wideband (UWB) antenna. (Note, all other parameters are given in Table 1.).

Parameters	Dimensions (mm)
Radius of hexagonal slots	1.25
Width of hexagonal slots	0.25
Radius of via-holes	0.25
Height of via-holes	0.85

**Table 3:** Geometrical parameters of the MTM inspired UWB antenna loaded with EBG slots. (Note, all other parameters are given in Table 1 and Table 2).

Parameter	Dimensions (mm)
Radius of EBG slots	0.2
Gap between EBG slots	0.5

**Table 4:** Comparison with state-of-the-art UWB notched band antennas.

**References**	**Antenna Size (mm^3^)**	**No. of EBGs**	**Bandwidth of each EBG (GHz)**	**Controllable EBG Freq.**	Design Complexity
[3]	16 × 25 × 1.52	Single	0.71	Yes	No
[19]	44 × 44.4 × 0.1	Dual	0.21 and 0.21	No	No
[20]	48 × 50 × 1	Single	0.97	Yes	No
[21]	50 × 50 × 1.575	Dual	0.24 and 0.48	No	No
[22]	28 × 18 × 0.8	Dual	0.54 and 0.53	No	Yes
[23]	38 × 42 × 0.5	Single	0.76	No	Yes
[24]	8.5 × 22 × 0.8	Single	1.34	No	Yes
Proposed Work	20 × 20 × 0.87	Dual	1.14 and 1.41	Yes	No
